# Detection of corneal pathology among Indians using WBC count as inflammatory marker

**DOI:** 10.6026/973206300200478

**Published:** 2024-05-31

**Authors:** Susmitha Joshy, MC Chaitra

**Affiliations:** 1 Department of Ophthalmology, Sri Devaraj Urs Medical College, Tamaka Kolar, Karnataka, India; 2 Department of Ophthalmology, Sri Devaraj Urs Medical College, Tamaka Kolar, Karnataka, India

**Keywords:** WBC, corneal pathology, inflammatory marker

## Abstract

The multifaceted role of NLR as a biomarker in corneal pathologies, aiming to enhance clinicians' understanding for better patient outcomes is of interest. An extensive ophthalmic assessment was conducted. Patients with corneal pathologies were identified as cases and those with healthy cornea as controls. A complete WBC blood count was performed using Automated Flow Cytometric method and the counts of white blood cells, neutrophils, platelets, and lymphocytes where recorded. NLR, PLR, and MLR were calculated by dividing the Neutrophil/Platelet/Monocyte counts by the lymphocyte counts. The study revealed that the Neutrophil-to-Lymphocyte Ratio (NLR), Monocyte-to-Lymphocyte Ratio (MLR), and Platelet-to-Lymphocyte Ratio (PLR) were significantly higher in the case group compared to the control group. N/L proved the best predictor among inflammatory markers, followed by M/L and P/L, highlighting the intricate immune response in corneal diseases, urging customized assessments in ocular health research.

## Background:

The Neutrophil-to-Lymphocyte Ratio (NLR), a derived biomarker from the simple ratio of neutrophil to lymphocyte counts in peripheral blood, encapsulates both the innate response led by neutrophils and adaptive immunity supported by lymphocytes [1]. Neutrophils, crucial in the initial immune response against pathogens, engage in mechanisms like chemotaxis, phagocytosis, and cytokine production. Additionally, neutrophils act as primary effectors during the systemic inflammatory response (SIRS), regulating adaptive immunity. Elevated NLR, observed in conditions like infection, stroke, myocardial infarction, trauma, cancer, surgery complications, and tissue damage-induced SIRS, signifies an imbalance marked by increased neutrophils and reduced lymphocytes The pro inflammatory state during the early hyper dynamic phase of infection, mediated by neutrophils, contributes to this NLR elevation. SIRS, associated with suppressed neutrophil apoptosis, intensifies neutrophil-mediated innate responses, accentuating the characteristic rise in NLR Corneal pathology encompasses a diverse range of disorders, including infections, inflammatory conditions, and degenerative diseases, posing threats to vision and ocular health. White blood cell (WBC) markers, particularly the NLR, play a crucial role in identifying, monitoring, and managing these conditions, offering valuable insights into the underlying inflammatory processes within the cornea. Eye disorders generally involve some degree of inflammatory burden [2]. However, uncertainties persist in this relationship due to limited sample sizes in existing studies, especially in the context of retinal vein occlusion, age-related macular degeneration, diabetic retinopathy and corneal pathologies [3, 4, 5]. While studies often focus on specific WBC markers, such as NLR, for their established significance in indicating inflammation and immune response, it's crucial to acknowledge the diversity within the white blood cell population. Various subtypes, including neutrophils, lymphocytes, monocytes, eosinophils, and basophils, each play unique roles in the immune response, offering valuable insights into different aspects of inflammation and disease progression. Therefore, it is of interest to unravel the implications of an elevated NLR, MLR and PLR for patients with eye diseases, providing clinicians with insights for early interventions and improved outcomes.

## Materials and Methods:

This meticulously conducted prospective case-control study received ethical approval from the institutional committee. Study conducted over a period of 15 months sample size included 60 cases and 60 controls.

Inclusion criteria involved patients who provided informed consent and were diagnosed with various corneal pathologies, designated as cases. Controls consisted of individuals with healthy corneas.

The exclusion criteria for this study encompassed individuals meeting the following conditions:

[1] Having a prescription history of tropical or systemic immunosuppressant and hormone medication within the past three months,

[2] Undergoing ocular surgeries (cataract surgery, corneal surgery, conjunctiva surgery, lacrimal canal surgery, and tear gland surgery) in the recent three months,

[3] Having a history of corneal contact lens wearing within three months,

[4] Experiencing conditions such as glaucoma, diabetic retinopathy (DR), retinal vein occlusion (RVO), thyroid-associated ophthalmopathy (TAO), and age-related macular degeneration (AMD), and

[5] Suffering from diabetes, cardiovascular disease, acute/chronic infections, autoimmune diseases, haematological diseases, and malignant tumors.

[6] History of fever, URTI, LRTI.

These exclusion criteria were established to ensure a focused and specific participant group for the study, minimizing potential confounding factors and enhancing the reliability of the results. The ophthalmic assessment involved critical examinations, including visual acuity assessment, slit lamp biomicroscopy, pachymetry, Tomey EM 4000 specular microscopy, anterior segment OCT, and fundoscopy. Cases with corneal pathologies and controls with healthy corneas were identified. Peripheral venous blood samples were collected for a complete blood count (CBC) using Automated Flow Cytometric methods. Counts of white blood cells, neutrophils, platelets, and lymphocytes were recorded. All patients in cases and controls had normal WBC counts. The Neutrophil-to-Lymphocyte Ratio (NLR), Platelet-to-Lymphocyte Ratio (PLR), and Monocyte-to-Lymphocyte Ratio (MLR) were calculated based on these counts. Data analysis employed SPSS 22 version software, presenting categorical data as frequencies and proportions, with the Chi-square test determining significance. Continuous data were represented as mean and standard deviation, and the independent t-test identified mean differences between groups. This methodologically rigorous study provides a comprehensive exploration of corneal pathologies, integrating clinical assessments and comprehensive blood analyses for a holistic understanding of the subject matter.

## Results:

**Figure 1:**
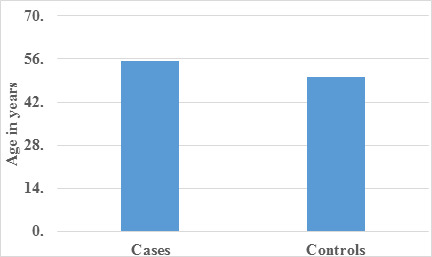
Graph showing Comparison of mean age among case and control

**Figure 2:**
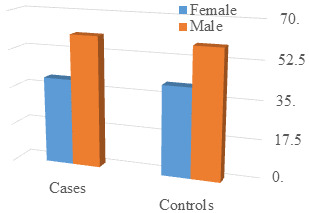
Graph showing Distribution of subjects according to sex among cases and controls

**Figure 3:**
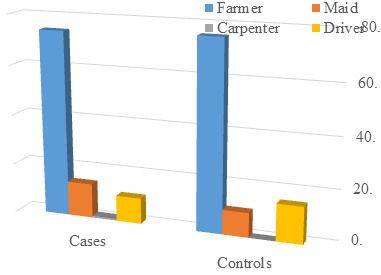
Graph showing Distribution of subjects according to occupation among cases and controls

**Figure 4:**
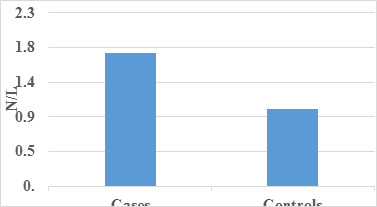
Graph showing comparison of Mean N/L among cases and controls.

**Figure 5:**
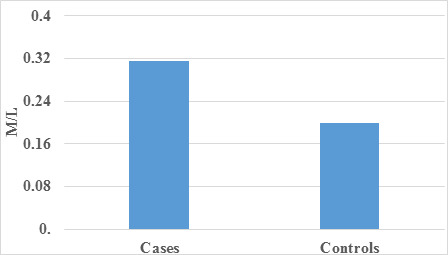
Graph showing Comparison of mean M/L among case and control

**Figure 6:**
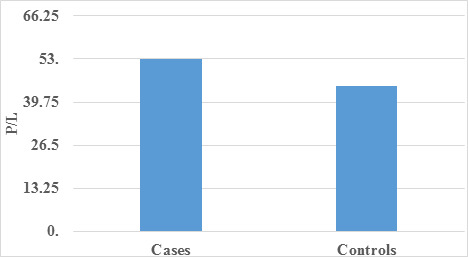
Graph showing Comparison of mean P/L among case and control

Data of 120 patients were analyzed. In the comparative analysis of mean age between the case and control groups (Table 1), a statistically significant difference was evident, indicating a notable divergence in age distribution. This observation was visually depicted in Figure 1, emphasizing the distinct mean age values for the two groups through graphical representation. Moving on to the examination of gender distribution (Table 2), the p-value of 1.00 suggested no statistically significant difference between cases and controls concerning sex. This information was graphically portrayed in Figure 2, providing a clear visual representation of the gender composition in both groups. Similarly, the distribution of subjects based on occupation (Table 3) yielded a p-value of 0.591, denoting no significant difference between cases and controls in terms of occupation. Figure 3 complemented this finding with a graphical illustration of occupation distribution. Subsequently to the analysis of inflammatory markers, specifically Neutrophil-to-Lymphocyte Ratio (N/L), Monocyte-to-Lymphocyte Ratio (M/L), and Platelet-to-Lymphocyte Ratio (P/L) (Table 4, Table 5-Table 6), revealed statistically significant differences between case and control groups. Notably, N/L emerged as the superior predictor among the three parameters, followed by M/L, while P/L exhibited the least predictive power. These findings highlight the potential of N/L as a key biomarker in gauging the inflammatory status associated with corneal conditions (Table 7-Table 8). The graphical representations(Figure 4, Figure 5-Figure 6) provide a visual summary, enhancing the comprehensive understanding of the observed differences between the groups. In conclusion, this detailed analysis contributes valuable insights into the relationship between demographic factors, inflammatory markers, and corneal pathologies.

## Discussion:

Neutrophils and lymphocytes, integral components of the immune system, play distinct roles. Neutrophils, representing innate immunity, contribute to the initial defence by producing chemokine, cytokines, vascular endothelial growth factor, and matrix metalloproteinase. On the other hand, lymphocytes, part of adaptive immunity, finely regulate specific immune responses [6]. The interaction between neutrophils and lymphocytes influences the amplitude of the immune response. Elevated neutrophil numbers have been associated with decreased lymphocyte activity [7, 8- 9]. Recently, the Neutrophil-to-Lymphocyte Ratio (NLR) has gained prominence as a systemic inflammation indicator, proving valuable in various disorders, including eye diseases. NLR serves as an independent prognostic biomarker, predicting significant outcomes in diverse clinical settings, such as mortality, morbidity, and long-term survival. The study employs a comprehensive approach to investigate the impact of age, gender, occupation, and inflammatory markers (N/L, M/L, P/L) on corneal pathologies. The observed statistical significance in mean age differences between cases and controls suggests age's pivotal role in the study. Figure 1 likely portrays this divergence visually, aiding in the identification of potential age-related confounding factors. The lack of statistical significance in gender distribution (P Value 1.00) underscores gender compositional similarity in cases and controls. Figure 2, potentially displaying gender distribution through bars or pie charts, becomes a cornerstone in eliminating gender-related biases that could impact the results of other variables. The non-significant difference in occupation (P Value 0.591) suggests occupational status might not confound study outcomes. Figure 3, likely illustrating the distribution of different occupations in both groups, aids in assessing population representativeness and the necessity of occupation as a controlled variable. The observed statistical significance in mean N/L, M/L, and P/L between cases and controls implies these parameters are crucial discriminators. Figure 4, Figure 5, and Figure 6 visually illustrate these differences. The nuanced statement that "N/L is a better predictor followed by M/L, and P/L is the least" adds depth, suggesting N/L as a potential key biomarker, enriching the understanding of corneal pathologies. The study's findings reveal an association between inflammatory markers, specifically Monocyte-to-Lymphocyte Ratio (MLR), Neutrophil-to-Lymphocyte Ratio (NLR), and Platelet-to-Lymphocyte Ratio (PLR), in corneal pathologies compared to healthy controls. Their capacity to induce pro-inflammatory cytokines and adhesion molecules underscores their importance in immune responses. This sets the foundation for understanding how imbalances in these cell types may contribute to corneal pathologies. The recognition of NLR as an indicator of systemic inflammation aligns with existing literature. The study draws parallels with a previous investigation by Emine *et al.* indicating elevated NLR in keratoconus patients and its correlation with disease severity [10].This not only supports the current findings but also establishes consistency across different studies. The release of inflammatory mediators by platelets is highlighted, indicating their crucial involvement in the inflammatory cascade. The mention of a study by Oltulu R, which reports a low level of MLR in keratoconus patients signifying a non-inflammatory disorder of the cornea, introduces a nuanced perspective [11- 12]. This divergence in findings underlines the complexity of corneal disorders and the need for further research to reconcile contradictory results. MLR can be used for Understanding the chronicity of the inflammatory response is deemed crucial in comprehending disease progression and determining appropriate treatment strategies. The advantages of using NLR, PLR, and MLR as inflammatory markers, citing their easy availability, stability, and low cost. This supports the practicality and feasibility of incorporating these markers into clinical assessments and research studies. In summary, this study contributes valuable insights into the association between inflammatory markers and corneal pathologies. The discussion not only elucidates the significance of specific ratios but also places them within the broader context of the inflammatory response, providing a foundation for future research and potential clinical applications.

## Limitations:

The study offers valuable insights into inflammatory markers related to corneal conditions, yet acknowledges limitations. These include the absence of causal inference from the identified elevated NLR, MLR, and PLR in the case group. Single-center focus may limit generalizability, introducing selection bias.

## Conclusion:

The study highlights differences in NLR, MLR, and PLR between case and control groups, with all ratios higher in cases, indicating increased inflammation in corneal conditions. N/L emerges as the superior predictor, followed by M/L and P/L, underscoring NLR's significance in assessing inflammatory status. Tailored treatment strategies may be warranted based on specific inflammatory profiles, with NLR guiding therapy targeting neutrophil or lymphocyte regulation. Monitoring changes in these ratios over time informs treatment adaptation and predicts disease progression in corneal conditions. Elevated NLR, MLR, and PLR imply systemic inflammation in corneal disorders, suggesting inflammation-targeted therapies may be beneficial. Further research into immune cell mechanisms could unveil precise therapeutic targets for improved interventions, enhancing understanding and management of corneal pathologies.

## Figures and Tables

**Figure 7:**
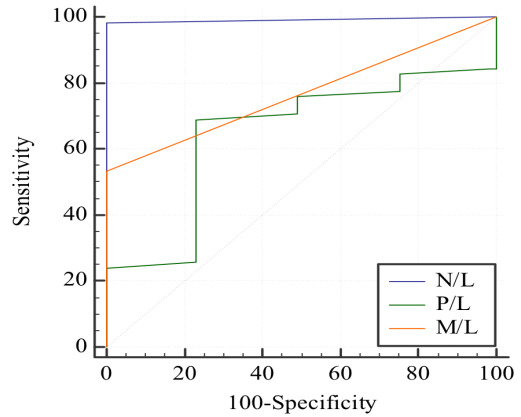
Graph showing ROC plot of the various markers.

**Table 1:** Comparison of mean age among case and control

	**Mean**	**Std. Deviation**	**P Value**
Cases	55.28	10.486	0.002
Control	50.11	6.897	
There was a statistically significant difference found between case and control with respect to age

**Table 2:** Distribution of subjects according to sex among cases and controls

	**Cases**		**Controls**	
	**N**	**%**	**N**	**%**
Female	23	39.70%	25	41.00%
Male	35	60.30%	36	59.00%
P Value 1.00, there was no statistically significant difference found between cases and controls with respect to gender.

**Table 3:** Distribution of subjects according to occupation among cases and controls

	**Cases**		**Controls**	
	**N**	**%**	**N**	**%**
Carpenter	1	1.70%	0	0.00%
Driver	6	10.30%	9	14.80%
Farmer	43	74.10%	46	75.40%
Maid	8	13.80%	6	9.80%
P Value 0.591, there was no statistically significant difference found between cases and controls with respect to occupation

**Table 4:** Comparison of mean N/L among case and control

	**Mean**	**Std. Deviation**	**P Value**
Cases	1.73	0.714	<0.001
Control	1	0	
There was a statistically significant difference found between case and control with respect to N/L

**Table 5:** Comparison of mean M/L among case and control

	**Mean**	**Std. Deviation**	**P Value**
Cases	0.316	0.2016	<0.001
Control	0.2	0	
There was a statistically significant difference found between case and control with respect to M/L

**Table 6:** Comparison of mean P/L among case and control

	**Mean**	**Std. Deviation**	**P Value**
Cases	52.93	15.83	<0.001
Control	44.73	9.82	
There was a statistically significant difference found between case and control with respect to P/L

**Table 7:** corneal pathologies

		**Cases**	
		**N**	**%**
RE CORNEA	ARCUS	10	17.20%
	CLEAR	11	19.00%
	Corneal degenerations	10	17.20%
	Cornel dystrophy	1	1.70%
	Epithelial defect	5	8.60%
	Healed corneal ulcer	1	1.70%
	Macular grade corneal opacity	2	3.40%
	Nebular grade corneal opacity	18	31.00%
LE CORNEA	ARCUS	3	5.20%
	Clear	25	43.10%
	Corneal degeneration	8	13.80%
	Corneal dystrophy	2	3.40%
	Epithelial defect	6	10.30%
	Healed corneal ulcer	1	1.70%
	Nebular grade corneal opacity	13	22.40%

**Table 8:** Showing average AUC and CI for the various markers

	**AUC**	**95%CI ^b^**
N/L	0.991	0.954 to 1.000
M/L	0.767	0.681 to 0.840
P/L	0.648	0.555 to 0.733
N/L is better predictor among three parameters followed by M/L and P/L is the least among three
